# ‘‘Two-Week Waits”—Are They Leading to Earlier Diagnosis of Soft-Tissue Sarcomas?

**DOI:** 10.1155/2010/312648

**Published:** 2010-09-26

**Authors:** W. St. J. Taylor, R. J. Grimer, S. R. Carter, R. M. Tillman, A. Abudu, L. Jeys

**Affiliations:** The Royal Orthopaedic Hospital, Bristol Road South, Birmingham B31 2AP, UK

## Abstract

*Introduction*. The ‘‘two-week wait” was established as a potential means of diagnosing malignant tumours earlier. This paper investigated whether these clinics are leading to earlier diagnosis of malignant soft-tissue lumps. *Method*. We identified all referrals to our centre from a database over a 4-year period. *Results*. 2225 patients were referred to our unit for investigation of a soft-tissue mass. 576 (26%) were referred under the ‘‘two-week wait” criteria. 153 (27%) of which were found to have a malignant or borderline malignant diagnosis. 1649 patients were referred nonurgently. 855 (52%) of which were diagnosed with a malignant or borderline lesion. The average size at diagnosis was 9.4 cm with no difference in size between the different referral routes. *Conclusion*. There is little evidence that the two-week wait clinic is leading to earlier diagnosis of soft-tissue sarcomas with the majority still being referred nonurgently.

## 1. Introduction

The Department of Health set up the “two-week wait” directive after concerns of diagnostic delay in patients with suspicion of cancer [1]. The directive requires that patients should be seen at a referral centre within two-weeks and that a diagnosis should be obtained within 31 days of referral. For those with proven malignancy treatment should then commence within a further 31 days. 

 There has been considerable controversy about the benefits of two-week wait clinics with some claiming that they will not improve the diagnosis of patients with malignancy and others that they will delay diagnosis and management of other nonmalignant conditions. The diagnostic yield of malignancy has also varied widely from 9.4% to 16.4% for bowel and breast cancer, respectively, [2, 3]. One study looking into urological malignancies [4] found that 11 out of 35 patients (31.4%) referred with a high PSA had prostate cancer, but most cases were beyond cure. It also found that only one of 19 patients (5.3%) referred with a testicular lump had cancer. They concluded that the scheme as a whole was unlikely to improve cancer outcomes for urological cancers. There remains uncertainty of the value of two-week wait clinics in expediting the diagnosis of cancer. 

 Only one paper has thus far reported on the efficiency of these clinics for sarcoma patients, demonstrating that 10 out of 40 patients referred had a malignant tumour (25%). However, less than 7% of all referrals to that unit came through this system and most of the patients diagnosed had bone tumours [5]. 

 Soft-tissue sarcomas are a rare type of cancer with about 2000 cases a year in the UK [6]. Delays in diagnosis are frequent and the average size at diagnosis remains at about 10 cm [7, 8]. Guidance was first published in 1999 highlighting worrying features that should prompt referral and this was reiterated in the next version of the guidance in 2005 [9, 10]. These criteria are now widely used to instigate a two-week referral for a suspected Soft-tissue sarcoma. 

 The aim of this paper was to investigate how effective the “two-week wait” system was in diagnosing malignancy in Soft-tissue lumps and what proportion of patients eventually found to have malignancy were referred through this route for Soft-tissue lesions. We also investigated whether as a result of a two-week referral patients were diagnosed earlier and thus whether their tumours were smaller.

## 2. Methods

Since 2005, the oncology database at our centre has been able to identify the type of referral for all patients referred to the unit. This includes the type of referral (i.e., routine or urgent under the two-week wait) and where the referral has come from (i.e., GP or hospital doctor). We analysed data from 1st November 2005 to 30th April 2009 on all patients referred to our Unit with a possible Soft-tissue sarcoma. 

 We identified details about the patients' diagnosis and the size of the tumour at diagnosis, their referral route, that is, who referred the patient and whether they were referred on purely clinical suspicion or whether they had had a biopsy or imaging prior to referral. Patients referred from the local region were identified by postcode and compared with those referred from outside the region. The final diagnosis was used to identify whether the patient did indeed have a Soft-tissue sarcoma or some other diagnosis.

## 3. Results

During the time period of this study, 364 patients were referred directly to our unit on clinical suspicion alone of a possible Soft-tissue sarcoma, without any prior investigation as per the guidelines. 48 of these patients (13%) had a malignant or borderline lesion with the remainder having a benign process or no abnormality. 

 Another 212 patients had undergone investigations prior to referral, either organized by their local hospital or by their GP which had raised the possibility of a sarcoma being present. Of these, 104 (49%) had a malignant or borderline lesion. 75 of the 104 had undergone imaging at their local hospital following a two-week wait referral there which had raised the suspicion of malignancy. 16 patients had been referred following imaging organized by their GP which had raised the suspicion of malignancy. 13 patients had been referred under the two-week wait following the unexpected diagnosis of sarcoma following excision of a lump, three by their GP and ten at their local hospital. 

 During the same time period 1649 patients were referred non urgently to our unit for investigation of a Soft-tissue lesion. In this group there were 855 malignant or borderline lesions of which 520 were Soft-tissue sarcomas, 83 were Soft-tissue cancers, 40 were bone sarcomas, 56 were non sarcoma bone cancers and 156 borderline lesions. 

 In total during the time period of this investigation, 610 Soft-tissue sarcomas were diagnosed of which only 90 (14.8%) were referred via the “two-week wait” and the other 520 (85.2%) via routine referral. 62 (10.2%) of the 610 Soft-tissue sarcomas were via GP referrals direct to the unit and 548 (88%) were tertiary referrals from other consultants. 

 The average size of all the Soft-tissue sarcomas was 9.4 cm. The mean size of those diagnosed following urgent referral under the two-week wait was 10.1 cm compared to 9.3 cm for those referred routinely (*P* = .28). However the average size of those referred directly to the unit by GPs under the two-week wait was 11 cm compared to 9.3 cm for those referred urgently under the two-week wait to other hospitals first (*P* = .18). 

 Overall 27.5% of the sarcomas were superficial to the fascia and the proportion that were superficial was virtually the same no matter what the referral route. 29% of the sarcomas referred by the urgent two-week wait were found to have a superficial STS compared with 27% detected through the non urgent route. 

 There was also no detectable difference in the location of the tumours diagnosed by the two different routes. Overall the most common site was the thigh where 35% of all the Soft-tissue sarcomas were located. Although 43% of the Soft-tissue sarcomas diagnosed by the two-week wait were in the thigh, 33% of those referred through other routes were also in the thigh (*P* = .21). There was no discernible difference in the size or site of the tumours when comparing where patients were referred from but most of the patients with subcutaneous sarcomas were referred from the West Midlands.

## 4. Discussion

The aim of this paper has been to investigate the effectiveness of the two-week wait clinic in diagnosing malignancy in Soft-tissue lumps. Of patients referred directly to our unit by local GPs, using clinical criteria alone, 13% had a malignant or borderline lesion. If the patient had imaging or biopsy suggestive of malignancy prior to referral, the diagnostic rate not surprisingly increased to 49%.

 One of the major findings of this study however was that the “two-week wait” clinics only picked up 90 out of 610 Soft-tissue sarcomas that were diagnosed (14.8%), with the remainder being referred by other routes. There was only a minimal difference in size between the tumours at diagnosis, no matter how they were referred, which is very disappointing. The overall size of 9.4 cm is still considerably in excess of other series, for example the Scandinavian Sarcoma Group quote an average size of around 7 cm and a recent paper from Italy quotes 6 cm [11, 12]. It is almost double the size indicated in the Guidance to prompt referral [9].

 The reasons for delays in diagnosis of Soft-tissue sarcomas have previously been explored and are part due to lack of both patient and doctor awareness, with considerable delays at every step of the pathway [7].

 Our data suggests that the majority of patients eventually found to have Soft-tissue sarcomas are still being referred non urgently to local hospitals for investigation. The reason for this is not clear but may be because of patient choice or GPs not considering the potential diagnosis of malignancy in lumps and bumps. This was one of the reasons why diagnostic clinics were recommended as part of the NICE guidance “Improving outcomes for patients with sarcomas” [13]. Each English Cancer Network has now defined where it's “diagnostic clinic” should be and this will hopefully allow a more rapid diagnostic pathway for this group. 

 There is no doubt however that there remains poor public awareness of the significance of Soft-tissue lumps. The importance of identifying breast lumps is well known and women are advised to regularly self-check and there is a national screening program. The average size of breast cancers now diagnosed is 2.1 cm compared with almost 10 cm for Soft-tissue sarcomas [14]. Earlier diagnosis when the tumour is smaller will lead to improved outcomes for patients with sarcomas [15, 16]. 

 The other interesting finding form this study was the high incidence of bony lesions identified even though the patients were referred ostensibly with a Soft-tissue lesion. Both benign and malignant lesions protruding from a bone (e.g., an osteochondroma or a chondrosarcoma) may simulate a Soft-tissue mass leading to the confusion. Clearly, in most cases an X-ray would clarify the nature of the underlying lesion and this may be considered to be a useful investigation to be carried out in cases of uncertainty, either by the GP or the diagnostic clinic. 

 This study has demonstrated that up until now the two-week wait referral system has made little difference to improve the diagnosis of Soft-tissue sarcomas. Patients are still being diagnosed late, when the tumours have reached a considerable size. Whether this is because of lack of patient or GP concern requires further investigation. The fact that so few patients with proven Soft-tissue sarcomas were referred through the very system designed to speed up the diagnostic pathway is also disappointing and suggests the need for increased GP awareness of the optimum pathway for early diagnosis. Further initiatives are needed to heighten awareness of the potential worrying features of Soft-tissue lumps and bumps and to identify the most specific factors that could lead to earlier diagnosis. As far as Soft-tissue sarcomas are concerned, the “two-week wait” pathway does not seem to have been helpful. The suggestion by Pencavel et al. that patients with suspicious lumps should be investigated by ultrasound prior to referral may have some merit but will rely very much on the quality of who is doing the scan [17].
